# Contrasting patterns of population structure and gene flow facilitate exploration of connectivity in two widely distributed temperate octocorals

**DOI:** 10.1038/hdy.2017.14

**Published:** 2017-03-15

**Authors:** L P Holland, T L Jenkins, J R Stevens

**Affiliations:** 1 Department of Biosciences, College of Life and Environmental Sciences, University of Exeter, Exeter, UK

## Abstract

Connectivity is an important component of metapopulation dynamics in marine systems and can influence population persistence, migration rates and conservation decisions associated with Marine Protected Areas (MPAs). In this study, we compared the genetic diversity, gene flow and population structure of two octocoral species, *Eunicella verrucosa* and *Alcyonium digitatum,* in the northeast Atlantic (ranging from the northwest of Ireland and the southern North Sea, to southern Portugal), using two panels of 13 and 8 microsatellite loci, respectively. Our results identified regional genetic structure in *E. verrucosa* partitioned between populations from southern Portugal, northwest Ireland and Britain/France; subsequent hierarchical analysis of population structure also indicated reduced gene flow between southwest Britain and northwest France. However, over a similar geographical area, *A. digitatum* showed little evidence of population structure, suggesting high gene flow and/or a large effective population size; indeed, the only significant genetic differentiation detected in *A. digitatum* occurred between North Sea samples and those from the English Channel/northeast Atlantic. In both species the vast majority of gene flow originated from sample sites within regions, with populations in southwest Britain being the predominant source of contemporary exogenous genetic variants for the populations studied. Overall, historical patterns of gene flow appeared more complex, though again southwest Britain appeared to be an important source of genetic variation for both species. Our findings have major conservation implications, particularly for *E. verrucosa*, a protected species in UK waters and listed by the IUCN as ‘Vulnerable’, and for the designation and management of European MPAs.

## Introduction

Population connectivity has emerged as a key factor in the sustainable management of marine resources (Fogarty and Botsford, 2007; Da Silva *et al.*, 2014), in tracking invasive species (Pérez-Portela *et al.*, 2012), in monitoring the effects of climate change (Munday *et al.*, 2009; Gerber *et al.*, 2014), and in designating networks of protected areas (Jones *et al.*, 2007; Marti-Puig *et al.*, 2013). For most benthic marine organisms, connectivity is typically defined by dispersal during early life stages and is intimately associated with oceanic currents and topographical features (Cowen *et al.*, 2007). However, connectivity can vary across marine taxa, even between closely related species over similar spatial scales (Bargelloni *et al.*, 2003, 2005; Charrier *et al.*, 2006; Kool *et al.*, 2013) and population structure can be determined by the extent of dispersal from distant vs local sources, resulting in fully ‘open’ (panmictic) to fully ‘closed’ (isolated) populations (see Cowen and Sponaugle, 2009 and references therein). Perhaps most importantly from an applied perspective, population structure and gene flow can be used as a proxy for understanding population connectivity (Hedgecock *et al.*, 2007; Lowe and Allendorf, 2010; Kool *et al.*, 2013).

Advances in our knowledge of marine population connectivity are fundamental for the strategic allocation of available resources in a way that maximises protection of marine biodiversity (Kool *et al.*, 2013). Moreover, the global extent of protected areas is unlikely to mitigate the current rate of marine and terrestrial biodiversity loss (Mora and Sale, 2011). Among the 15 European countries that have signed the Oslo/Paris (OSPAR) Convention (for the protection of the marine environment of the northeast Atlantic), there is a requirement to establish an ‘ecologically coherent’ network of Marine Protected Areas (MPAs), which collectively aims to deliver more benefits to biodiversity than single, unrelated MPAs (OSPAR Convention, 2013). As connectivity is a key feature of an MPA network, it is important that empirical estimates of population connectivity are considered during the designation or review stages of a network (Jones *et al.*, 2007). For example, guidelines for incorporating connectivity into designing networks of marine reserves are available for coral reefs and are likely to be useful for the management and protection of these systems (Almany *et al.*, 2009; McCook *et al.*, 2009). Several analyses of connectivity in established networks have also identified deficiencies that may reduce the efficacy of a network. For example, Puckett *et al.* (2014) modelled dispersal of the eastern oyster (*Crassostrea virginica*) on the Atlantic coast of North Carolina and showed that if marine reserves were too small – relative to the mean dispersal distance of the oyster– local retention of larvae was reduced; likewise, if reserves were spaced too far apart, connectivity became limited. While early landmark studies of genetic connectivity in the marine environment (for example, Palumbi, 2003) focused largely on gene flow, barriers to gene flow and isolation by distance (IBD), more recent studies (for example, Arizmendi-Mejía *et al.*, 2015; Gagnaire *et al.*, 2015) have further refined our understanding of drivers of marine genetic connectivity and have demonstrated the importance of additional factors in driving or disrupting genetic connectivity, for example, effective population size and genetic drift. Overall, such findings suggest a greater understanding of population structure and connectivity is required to optimise the conservation of marine biodiversity and to maximise the efficacy of such networks (for example, OSPAR Commission, 2006; Jones *et al.*, 2007)

Currently, and until such time as a robust understanding of the functioning of networks of MPAs is achieved, individual MPAs are typically designated based on the presence of rare or protected species or guilds of species; for example, in the waters of southwest Britain, the presence of *Eunicella verrucosa* (the pink sea fan) is often listed as a factor in the designation of an area as a Marine Conservation Zone (MCZ). However, population genetic studies of octocorals across this area, and the northeast Atlantic in general, are limited. Previous research in this region has had either a phylogenetic (for example, McFadden and Hutchinson, 2004) or phylogeographic focus (for example, Herrera *et al.*, 2012), while existing connectivity research on this subclass in the region has assessed the genetic diversity and structure of primarily Mediterranean species, for example, *Corallium rubrum* (Costantini *et al.*, 2007; Ledoux *et al.*, 2010; Aurelle *et al.*, 2011), *Eunicella singularis* (Costantini *et al.*, 2016), *Eunicella cavolini* (Masmoudi *et al.*, 2016) and *Paramuricea clavata* (Mokhtar-Jamai *et al.*, 2011; Arizmendi-Mejía *et al.*, 2015). As a result, genetic diversity and connectivity in this group remains understudied.

*Eunicella verrucosa* is an IUCN red-listed octocoral. It can be found from Angola to western Ireland, but its range in the British Isles is limited to southwest England, southwest Wales, and southern and western Ireland (Hayward and Ryland, 1995). In Britain, it is considered rare due to its limited distribution beyond the southwest (Hiscock *et al.*, 2010), although where it is found it can be relatively abundant and may form ‘forests’. Colonies are generally found inhabiting rocky substrates, at depths of 10–150 m, in areas of high turbidity with moderate to high current flow. *E. verrucosa* has an important role for the functional ecology of sublittoral ecosystems in which it occurs; it provides structural complexity and habitat for numerous epifauna and, as such, may be considered to be an ecosystem engineer (Hall-Spencer *et al.*, 2007; Pikesley *et al.*, 2016). Colonies are also vulnerable to trawling activity and, as a result, the designation of several MPAs across Britain includes *E. verrucosa* as a specific factor (a ‘protected feature’) in their designation.

*Alcyonium digitatum* (‘dead man’s fingers’) has a ubiquitous presence along rocky upper and circalittoral zones, typically to a depth of 200 m, and it can be found around most British and Irish coasts (Hayward and Ryland, 1995); it is represented in several MPAs across the UK network. It is widely distributed across the North Atlantic, ranging from Portugal to Norway, to eastern Canada, south to Cape Hatteras in the USA (Hartnoll, 1975; Watling and Auster, 2005). It is not a protected species, however, it is locally depleted in some areas by benthic trawling (Hinz *et al.*, 2011). Both species are thought to be lecithotrophic, gonochoristic (separate sexes) and broadcast spawners, with limited reports of hermaphroditism in *A. digitatum*; asexual reproduction may also be possible in *E. verrucosa* as genets can proliferate via fragmentation (Hartnoll, 1975; McFadden *et al.*, 2001; Munro, 2004). *Alcyonium digitatum* releases gametes in winter (December–January) and pelagic larvae can survive up to 14 weeks and beyond (Hartnoll, 1975). Spawning of *E. verrucosa* occurs towards the end of summer (August–September), though its pelagic larval duration is unknown (Munro, 2004). Studying patterns in genetic connectivity and assessing genetic diversity offers an alternative approach by which to infer the dispersal capabilities of these species.

In this study, two panels of microsatellites (Holland *et al.*, 2013a, 2013b) were used to assess the population structure and genetic connectivity of *E. verrucosa* and *A. digitatum* around the British Isles and northeast Atlantic. Specifically, we addressed the following questions: (i) what is the genetic diversity of each species and is it uniform across the sampling range; (ii) do both of these species show population genetic structure indicative of departures from panmixia; and (iii) what are the levels of gene flow and effective population size for each species? Finally, we consider the conservation and potential management implications of our findings for these species, both in terms of connectivity between existing MPAs and with regard to the designation of future MPAs.

## Materials and methods

### Study sites and sampling

**Figure 1:**
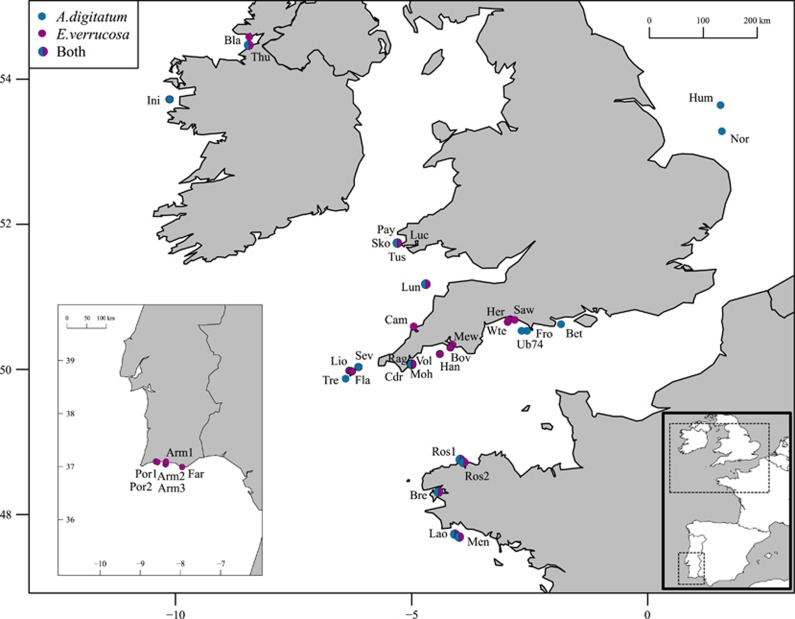
Map of the sites sampled in the northeast Atlantic. Pink circles represent sites where only *Eunicella verrucosa* were collected and blue circles represent where only *Alcyonium digitatum* were collected. Circles containing both colours represent sites in which both *E. verrucosa* and *A. digitatum* were collected. See Table 1 for details on population codes, sample size and latitude and longitude.

Samples of *E. verrucosa* (*N*=922) were collected from 27 sites ranging from southern Portugal to northwest Ireland, including sites around Brittany in northwest France, Lyme Bay in southern England and southwest Wales (Table 1 and Figure 1). The area sampled represents much of the northern range of the species. Samples of *A. digitatum* (*N*=655) were collected from 20 sites across a similar geographic area (with the exception of southern Portugal, where the species was not found); samples from two additional sites in the North Sea (Table 2 and Figure 1) were also included. The area sampled represents much of the southern range of *A. digitatum* in Europe. Samples of both species were collected between 2007 and 2012. The majority of samples were collected by SCUBA at depths between 10 and 35 m; additional samples of *A. digitatum* were collected by trawling (CEFAS scientific trawl, Lowestoft, UK). Colonies of *E. verrucosa* were sampled by removing a 3 cm terminal branch using sea-snips. This species is protected in UK waters, and all UK sampling complied with licenses granted by Natural England and the Marine Management Organisation (see Acknowledgements). Colonies of *A. digitatum* were sampled by removing a 1 cm^3^ section of tissue from a terminal thumb-like ‘branch’ using sea-snips. After removal, individual colonies were placed into mesh bags, brought to the surface, and quickly immersed in 95–100% ethanol for storage. In both species, samples were taken from individual colonies spaced at least 1 m apart to avoid sampling clones; previous studies of hard corals have identified potential clones at spatial scales up to 5 m apart (for example, Goffredo *et al.*, 2009; Foster *et al.*, 2012). This issue was also addressed after genotyping by identifying and excluding any duplicate genotypes occurring in the same population.

### DNA extraction and microsatellite genotyping

Total genomic DNA was extracted from ~10 to 20 polyps using a WizardR SV Genomics DNA Purification System kit (Promega, Southampton, UK) following the manufacturer’s protocol. Polyps were removed from colonies using forceps, or by using a scalpel to shave a portion of ~1 cm^2^ surface tissue from *A. digitatum* or 1–2 cm of coenenchymal tissue (excluding the gorgonin axis) from *E. verrucosa*. Microsatellites were amplified for both species and alleles were scored using GENEMAPPER v3.7 (Applied Biosystems, Paisley, UK). Full details of DNA extraction and microsatellite amplification conditions and multiplexing are given in a primer note for each species: *E. verrucosa* (Holland *et al.*, 2013a) and *A. digitatum* (Holland *et al.*, 2013b).

### Data screening and quality assessment

Duplicate genotypes were identified in CERVUS v3.0.3 (Kalinowski *et al.*, 2007) and were removed from further analyses. The presence of possible null alleles, allele scoring errors due to stuttering and large allele dropout was evaluated using MICRO-CHECKER v2.2.3 (Van Oosterhout *et al.*, 2004). Linkage disequilibrium and deviation from Hardy–Weinberg equilibrium (HWE) were tested in GENEPOP v4.2 (Rousset, 2008) using default parameters and the false discovery rate was used to detect type-1 errors (Storey and Tibshirani, 2003). To identify candidate markers under selection or linked with markers under selection, loci were screened using two different *F*_ST_ outlier detection methods in Lositan (Antao *et al.*, 2008) and Arlequin v3.5.2 (Excoffier and Lischer, 2010). Lositan assumes an island model and runs were conducted using the infinite alleles model. Parameters were set to 50 000 simulations, a 99% confidence interval and a false discovery rate of 0.1, with the neutral and forced mean *F*_ST_ enforced. In Arlequin, 50 000 simulations were run with 100 demes simulated per group and 10 simulated groups under the hierarchical island model. Samples were grouped by geographical region (*E. verrucosa*: Portugal, France, Ireland, Britain; *A. digitatum*: France, Ireland, Britain, North Sea) and results were considered significant if the *P*-value was <0.010.

### Genetic variation

Expected heterozygosity (*H*_exp_) and the inbreeding coefficient (*F*_IS_) for each population were estimated using the *diveRsity* package (Kennan *et al.*, 2013) in R (R Development Core Team, 2016). The *divBasic* function was used and *F*_IS_ significance was assessed using 95% confidence intervals using 1000 bootstrap replicates; the significance level for multiple comparisons was corrected using a Bonferroni correction (Dunn, 1961), which had the effect of slightly widening each interval. Allelic richness (*A*_r_) and private allelic richness (*PA*_r_) were calculated in HP-RARE v1.1 (Kalinowski, 2005) using a rarefaction method, which accounts for variation in sample size (each sample included a minimum of eight loci).

### Population structure

Population differentiation was analysed using pairwise *F*_ST_ (Weir and Cockerham, 1984) and *G*”_ST_ (Meirmans and Hedrick, 2011) measures using the *diffCalc* function in *diveRsity*, and significance was assessed as for *F*_IS_. To search for genetic structuring, a principal coordinates analysis (PCoA) was performed using a matrix of codominant genotypic genetic distances in GenALEx v6.5 (Peakall and Smouse, 2012). An analysis of molecular variance (AMOVA) was performed using Arlequin (10 000 permutations) to test for differentiation amongst geographical regions. Population structure was also analysed using a Bayesian clustering method: STRUCTURE v2.3.4 (Pritchard *et al.*, 2000), using a burn-in of 10^4^ and 10^6^ repetitions. An admixture ancestry model using population IDs as priors and correlated allele frequencies was chosen. To determine the number of populations (*K*), the delta *K* statistic (Evanno *et al.*, 2005) and the mean value of L(*K*) were examined in the POPHELPER R package (Francis, 2017). Ten replicate runs were aligned and merged in POPHELPER using CLUMPP and graphics were generated using the merged data. Initial runs for both species using *K* values 1–10 showed a very low likelihood for *K* values 6–10, therefore, subsequent runs included only *K* values of 1–5. A Mantel test was implemented in GenALEx to test whether any observed genetic structure was a product of IBD. Genetic distances were supplied as *F*_ST_/(1- *F*_ST_) matrices and were compared with the logarithm of geographic distances (km). Negative *F*_ST_ values were converted to zero for this analysis. Geographical distances were estimated in Google Earth by measuring the shortest in-water distance between sites in a straight line or by calculating the shortest distance following coastlines.

### Gene flow and effective population size

Contemporary (within the last few generations) and historical gene flow was estimated using two methods. Contemporary gene flow was analysed using BayesAss v3.0.4 (Wilson and Rannala, 2003), which estimates the fraction of immigrants in a population using Bayesian inference. Three runs were performed using 10^7^ iterations, a burn-in of 10^6^ and a sampling interval of 100, and an average of the gene flow estimates was calculated. The mixing parameters DeltaA, DeltaF and DeltaM were set to 0.10, 0.20 and 0.05 for *E. verrucosa*, and 0.30, 0.50 and 0.10 for *A. digitatum*, respectively. Convergence of the chains was validated using Tracer v1.6 (Rambaut *et al.*, 2014). Historical gene flow was calculated using the mutation-scaled migration rate *M* (*m*/μ where *m* is the immigration rate per generation) and the population parameter theta (4*N*_e_*μ) in Migrate-n v3.6 (Beerli and Felsenstein, 2001). Migrate-n is a coalescence-based program that has the benefit of providing values of immigration and emigration for each population and is therefore useful in scenarios of asymmetrical migration. A Brownian motion model was used and assumed a migration matrix with variable theta and estimated mutation rates for loci based on the data. A Bayesian likelihood strategy was initially run with default parameters to obtain start parameter estimates for theta and *M*. These parameters were supplied to the program in subsequent runs and the number of recorded steps was increased to 50 000. Prior uniform distributions for theta and *M* were set to min=0, max=100 and delta=10, and min=0, max=1000 and delta=100, respectively. To evaluate convergence of the chains, the effective sample size (>1000) and the shape of the histograms in the output files were examined.

Migrate-n was also used to calculate the mutation-scaled effective population size (*N*_e_). This was calculated from the optimum value of theta using the equation *N*_e_=theta/4 μ, assuming a microsatellite mutation rate (μ) of 10^−4^ per generation, as used in a previous study of a Mediterranean cup coral (Casado-Amezú *et al.*, 2012). Contemporary *N*_e_ was estimated using LDNE v1.31 (Waples and Do, 2008). The program was run assuming a model of random mating and the allowed frequency of observed alleles was set to 0.050.

## Results

### Data screening and quality assessment

For *E. verrucosa*, based on evidence of null alleles and significant deviation from HWE, one locus (Ever009) was omitted from the original microsatellite panel of Holland *et al.* (2013a). Five other loci also showed some deviation from HWE, however, departures from HWE occurred in only a few populations and these loci were retained. Similarly, linkage disequilibrium was detected in five populations, but each population showed different pairs of potentially linked loci. With no obvious trend in the pattern of linkage disequilibrium observed, this inconsistency was likely due to site-specific biological processes which we were not able to investigate further within this study; consequently, no loci were discarded on the basis of linkage disequilibrium and 13 were used for subsequent analyses. For *A. digitatum*, three loci (Adig003, Adig004 and Adig010) were discarded from the original microsatellite panel of Holland *et al.* (2013b) based on the presence of null alleles and significant deviations from HWE. Some evidence of linkage disequilibrium was also detected, but was minimal across populations and no further loci were omitted; eight loci were used for subsequent analyses.

A relatively low number of duplicate genotypes were identified in both species. In *E. verrucosa*, 17 individuals with duplicate genotypes were identified in nine samples (Table 1), while in *A. digitatum*, seven individuals with duplicate genotypes were identified in five samples (Table 2). Duplicates were removed from further analyses. The spread of duplicates across sites did not show any obvious pattern in either species, with the exception of a small sample of *E. verrucosa* (Cam) from north Cornwall, in which four duplicate individuals (across three genotypes) were identified out of a sample of only 11 individuals successfully genotyped.

*E. verrucosa* samples were monomorphic at several loci, but this was not consistent in all populations at the same locus. In comparison, *A. digitatum* was monomorphic at only one locus (Adig007) in three populations. For *E. verrucosa*, two loci (Ever013 and Ever014) were identified as outliers under the island model and one (Ever013) under the hierarchical island model (Supplementary Appendix 1). Accordingly, as both methods identified Ever013 as an outlier under positive selection, analyses of population structure, gene flow and effective population size excluded this locus; STRUCTURE, PCoA and BayesAss analyses were conducted using 13 loci as the assumptions of these methods are not violated by the inclusion of loci under selection (Pritchard *et al.*, 2000; Wilson and Rannala, 2003). One outlier locus (Adig006) was identified for *A. digitatum* by the island model, but not by the hierarchical island model (Supplementary Appendix 1); accordingly, eight loci were retained.

### Genetic variation

After removal of duplicate genotypes, genotypes of 905 individual specimens of *E. verrucosa* from 27 sites were analysed at 13 loci. For *A. digitatum*, genotypes of 648 individual specimens from 20 sites were analysed at eight loci. For *E. verrucosa*, measures of *H*_exp_ ranged from 0.367 (Black Rock) to 0.459 (Hand Deeps) and were generally consistent within regions, with minor differences between some regions (Table 1). A similar pattern was observed for *A*_r_, which ranged from 2.38 (Black Rock) to 2.79 (The Heroine Wreck); overall, both measures were slightly lower in the samples from Ireland. For *A. digitatum*, *H*_exp_ and *A*_r_ measures were also relatively uniform within and between regions (Table 2) and were consistently higher than for *E. verrucosa*; *H*_exp_ measures ranged from 0.594 (The Lucy Wreck) to 0.668 (Norfolk) and *A*_r_ ranged from 3.99 (Laonegued Taer) to 4.34 (Roscoff2). Private allelic richness (*PA*_r_) was also consistently higher for *A. digitatum* than for *E. verrucosa*; values for *A. digitatum* ranged from 0.075 (The Lucy Wreck) to 0.193 (Roscoff1), while values for *E. verrucosa* were between 0.001 (Camel Estuary) to 0.059 (Laonegued Taer). The majority of *F*_IS_ values for both species were positive; overall, however, few were significant, though generally at least one site in each region showed a significant positive *F*_IS_ coefficient (Tables 1 and 2). This finding suggested a deficiency of heterozygotes at some sites; for *E. verrucosa*, this was most apparent in several populations from Portugal, while both species showed significant, positive *F*_IS_ values at Roscoff2. The broader implications of these findings (inbreeding and/or a Wahlund effect caused by the inadvertent combining of data from separate populations) are discussed below. A small sample of *E. verrucosa* from the Camel Estuary (Cam) had a significantly negative *F*_IS_, indicating an excess of heterozygotes at this site.

### Population structure

Global *F*_ST_ and G”_ST_ measures across all populations of *E. verrucosa* were 0.012 and 0.023, respectively (Supplementary Appendix 2). In comparison, global *F*_ST_ and G”_ST_ measures for all populations of *A. digitatum* were lower (0.003 and 0.011, respectively) (Supplementary Appendix 2). For both species, global values were significantly different from zero.

For *E. verrucosa*, the largest significant pairwise *F*_ST_ (0.059) value was observed between Faro and the Camel Estuary, while the highest significant pairwise G”_ST_ (0.089) value observed was also between populations from Portugal and southwest Britain: Portamao2 and the Heroine Wreck (Supplementary Appendix 2). In contrast, the highest significant pairwise *F*_ST_ and G”_ST_ values (0.020 and 0.058, respectively) for *A. digitatum* were between populations from southwest Britain and the North Sea: Trenemene Reef and Norfolk (Supplementary Appendix 2). For both species, both pairwise measures were typically low and non-significant within regions and between populations from Britain and France. For *A. digitatum*, only pairwise comparisons with North Sea populations were significant. However, for *E. verrucosa*, many pairwise comparisons between Portugal populations and populations from Britain, Ireland and France were significantly different from zero.

**Figure 2:**
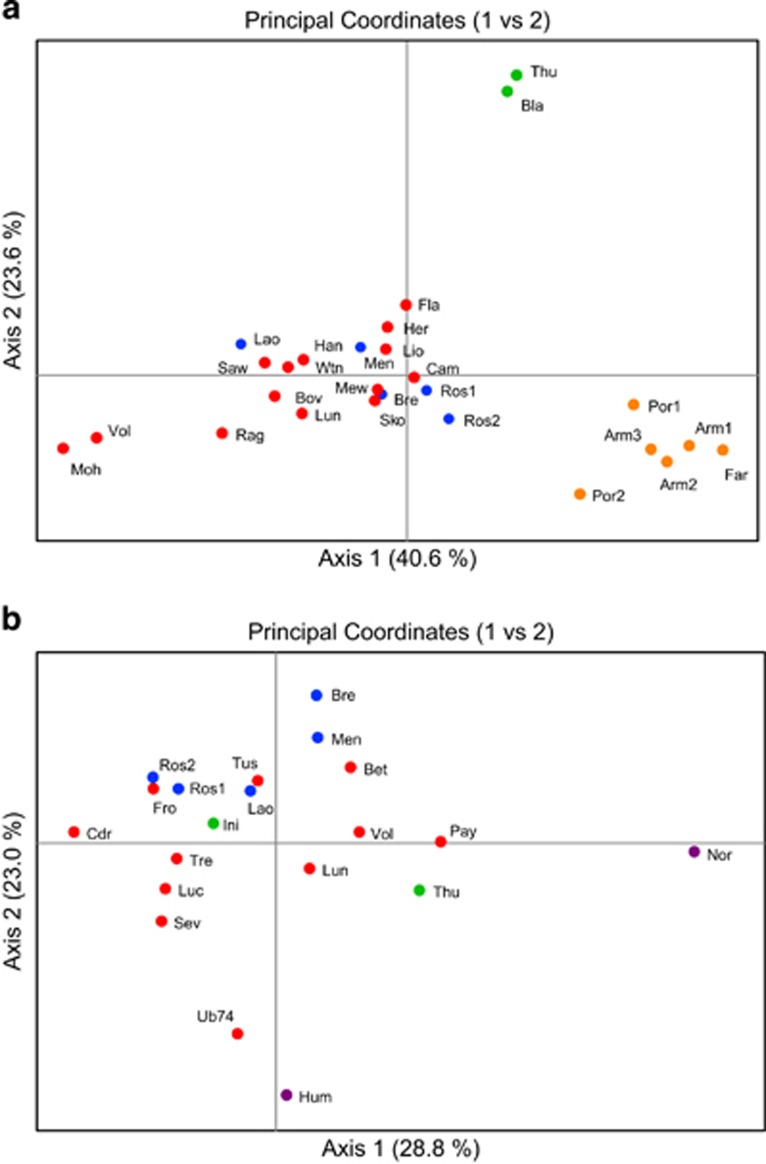
PCoA for *Eunicella verrucosa* (**a**) and *Alcyonium digitatum* (**b**). Colours correspond to regions: Britain (red), France (blue), Ireland (green), Portugal (orange), the North Sea (purple).

The PCoA suggested regional structure in *E. verrucosa* (Figure 2a), with evidence for three clusters: Portugal, Ireland, and populations from Britain and France. In contrast, little evidence of regional structure was apparent in *A. digitatum* (Figure 2b). There was some evidence for the isolation of the North Sea and UB74 Wreck populations of *A. digitatum*; however, genetic structure did not appear wholly concordant with geography, as the North Sea populations did not group together.

For the AMOVA, populations were grouped by geographical region for each species: Portugal, France, Ireland and Britain (*E. verrucosa*) and France, Ireland, Britain, and the North Sea (*A. digitatum*). In both species, global tests revealed that the majority of variation was explained by variation within populations (Supplementary Appendix 3). For *E. verrucosa*, a small but highly significant amount of variation was explained by differences between the geographical regions (*F*_CT_=0.016, *P*<0.001). Similarly, a significant (but much smaller) amount of variation was explained by differences between regions for *A. digitatum* (*F*_CT_=0.001, *P*=0.049).

**Figure 3:**
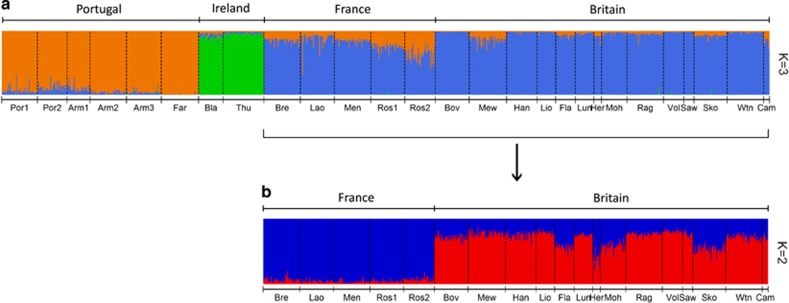
STRUCTURE analysis for *Eunicella verrucosa* using all populations (**a**) and hierarchical STRUCTURE analysis using populations from only Britain and France (**b**). The colours in the STRUCTURE plots correspond to genetic clusters, in which each individual is represented as a coloured vertical bar that represents that individual’s membership in each cluster. See Table 1 for details on population codes.

For *E. verrucosa*, both the mean L(*K*) and delta *K* statistics indicated *K*=3 as the most probable number of discrete populations within the data set (Supplementary Appendix 4). STRUCTURE analysis (Figure 3a) identified essentially the same groupings as observed in the PCoA (Figure 2a), but also indicated that all *E. verrucosa* colonies from France (and a few from Britain) shared some allelic similarities with *E. verrucosa* from Portugal. To explore potentially finer-scale population structure (<500 km distance between sites) in populations from Britain and France, a hierarchical STRUCTURE analysis was conducted using data from only these regions. The most likely number of populations was identified as *K*=2 (Supplementary Appendix 4), which revealed moderate structure partitioned between *E. verrucosa* populations from Britain and those from France, with some evidence of allelic variants more typical of *E. verrucosa* from France occurring in samples from Britain (Figure 3b). In contrast, for *A. digitatum*, the mean L(*K*) suggested panmixia (*K*=1; Supplementary Appendix 4). Analysis of delta *K* for *A. digitatum* suggested *K*=2; however, the delta *K* method is known to be unsuitable for accurately identifying *K* when *K*=1 (Evanno *et al.*, 2005).

**Figure 4:**
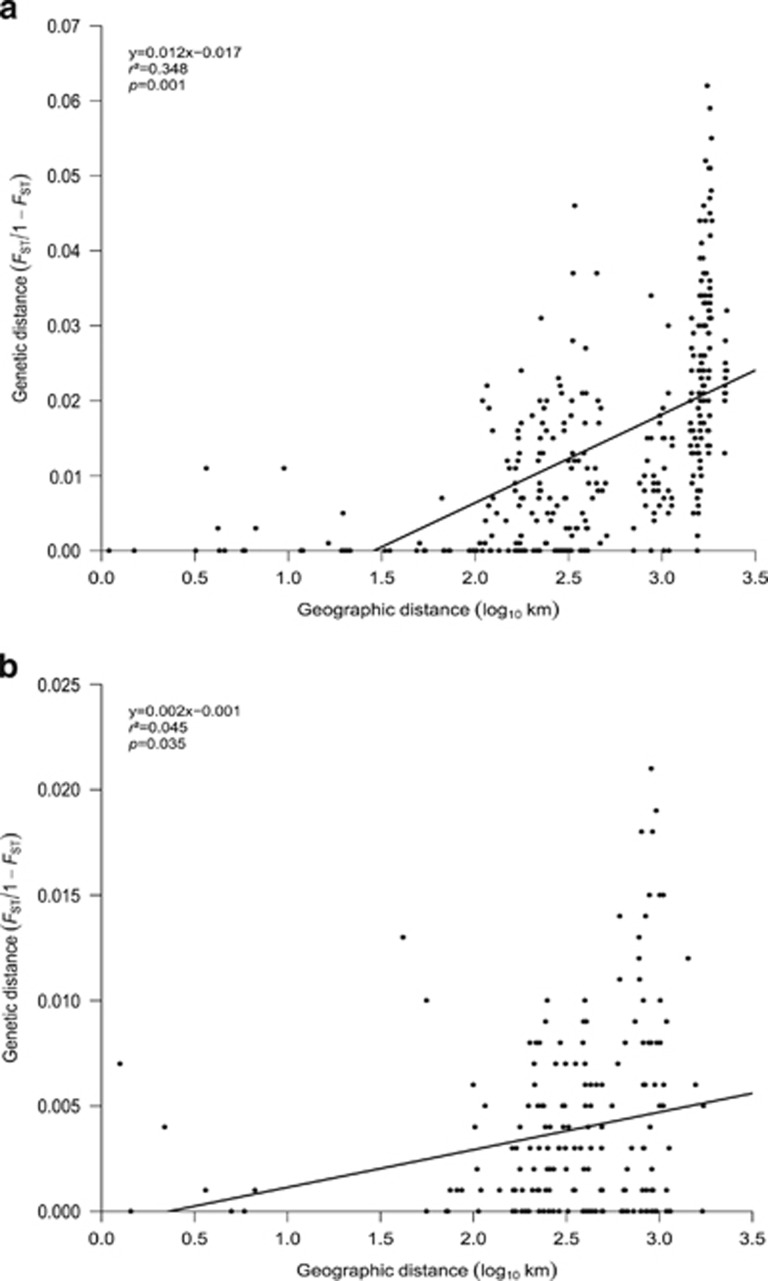
Relationship between genetic distance and geographic distance for *Eunicella verrucosa* (**a**) and *Alcyonium digitatum* (**b**).

Analysis of pairwise genetic and geographic distances between sample sites showed a moderate, significant correlation for *E. verrucosa* (*r*^2^=0.348, *P*=0.001; Figure 4a). The correlation was much weaker but remained significant when the samples from Portugal were excluded from the analysis (*r*^2^=0.083, *P*=0.004; Supplementary Appendix 5). Similarly, the correlation remained significant when the samples from Portugal and Ireland were excluded from the analysis (*r*^2^=0.077, *P*=0.003) (Supplementary Appendix 5). For *A. digitatum*, a weak, but similarly significant correlation between genetic and geographic distances was apparent (*r*^2^=0.045, *P*=0.035; Figure 4b); however, removal of the North Sea samples resulted in no correlation (*r*^2^<0.001, *P*=0.463; Supplementary Appendix 5). Analysis of both species was also carried out using *G*”_ST_ as the genetic distance; for *E. verrucosa* the result was similar to that obtained using *F*_ST_ (Supplementary Appendix 5), however, for *A. digitatum*, the correlation was lower and non-significant (Supplementary Appendix 5).

### Gene flow and effective population size

**Figure 5:**
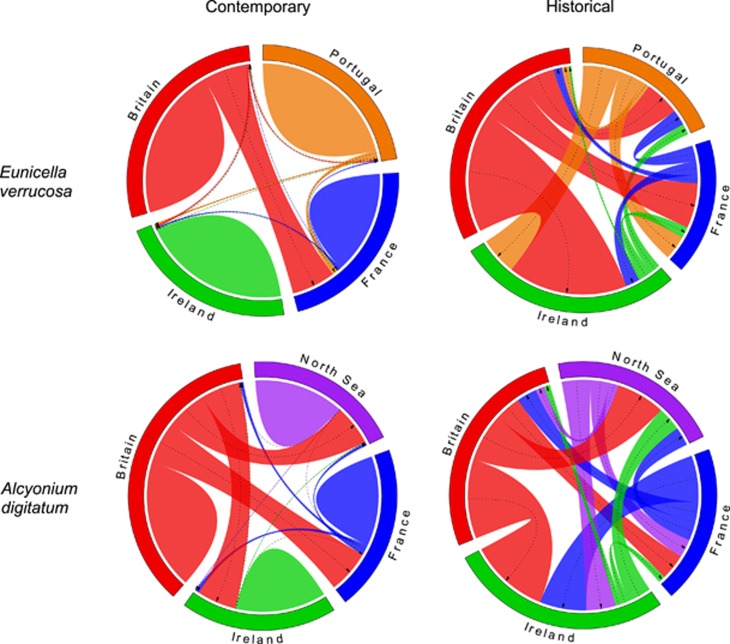
Gene flow diagrams for *Eunicella verrucosa* and *Alcyonium digitatum*. Contemporary gene flow estimates were derived from BayesAss and historical gene flow estimates were calculated using Migrate-n. Colours correspond to regions: Britain (red), France (blue), Ireland (green), Portugal (orange), the North Sea (purple). The direction of an arrow represents the direction of gene flow from one region to another. The width of the arrows denotes the relative amount of gene flow within the scenario being explored (that is, the wider the arrow, the more gene flow). The ‘humps’ in the estimates of contemporary gene flow represent gene flow originating from sample sites within regions. Patterns for each diagram are independent, that is, similar widths of arrows or humps do not represent the same amount of gene flow across each of the four diagrams (see Supporting Appendix 6 for exact gene flow estimates).

To estimate gene flow, samples of both species were classified by geographical region as per the AMOVA groupings. Contemporary gene flow estimates (using BayesAss) for both species indicated that the majority of gene flow originated from sample sites within regions (Figure 5). However, for both species, where some gene flow between regions was detected, populations in southwest Britain were the predominant source of exogenous allelic variants. For *E. verrucosa*, gene flow from Britain was predominantly into France, whereas in *A. digitatum* gene flow from southwest Britain into the North Sea, Ireland and France was observed. In comparison, contemporary gene flow into Britain appeared very limited for both species. For *E. verrucosa*, little genetic material was exchanged between Ireland and any other region; likewise, gene flow to/from Portugal was minimal, except for some minor gene flow from Portugal into France. Little or no gene flow from France was detected, suggesting that *E. verrucosa* in both France and Ireland are effectively sinks. In contrast, for *A. digitatum*, some gene flow from France to other regions was apparent, although gene flow from the North Sea and Ireland to other study areas was all but absent. For both species, estimates of historical gene flow (using Migrate-n) were somewhat more complex, with populations from Britain again acting as the main source of gene flow for both species, and with only limited gene flow into southwest Britain (Figure 5). Overall, historically, there appeared to have been considerably more gene flow between all regions.

Analyses of *N*_e_ were run using the same groupings as used in the gene flow analyses. Estimates of contemporary effective population sizes were infinite for both species (Supplementary Appendix 6). Historical effective population sizes for *E. verrucosa* indicated that samples from Britain had the largest *N*_e_, followed by those from Ireland, France and Portugal. In contrast, for *A. digitatum*, estimates of historical *N*_e_ in Ireland and the North Sea were by far the largest, being more than six times larger than the *N*_e_ for *E. verrucosa* in Britain. Estimates of *N*_e_ for *A. digitatum* from Britain and France were, in contrast, very small.

## Discussion

This study demonstrates that regional population structure is apparent in the octocoral species *E. verrucosa* sampled from sites around the northeast Atlantic, including northwest Ireland, southwest Britain, northwest France and southern Portugal. However, over a similar spatial area, another temperate octocoral, *A. digitatum*, showed only very limited population structure. Therefore, despite the similarities in habitat and life histories of these octocorals, patterns of genetic connectivity over approximately the same geographical area appear variable between species within Octocorallia. The implications of and possible causes for these apparent differences –differences in gene flow and/or effective population size– are now considered.

### Genetic diversity and inbreeding

Genetic diversity measures (*H*_exp_ and *A*_r_) were generally uniform across the sampling ranges of each species (Tables 1 and 2); however, higher estimates of both measures in *A. digitatum* indicated higher genetic diversity in this species than in *E. verrucosa*. In comparison to other studies of temperate corals (Table 3), the genetic diversity of *A. digitatum* observed in the current study was higher than or comparable to that reported in the octocorals *Eunicella singularis* (Costantini *et al.*, 2016) and *E. cavolini* (Masmoudi *et al.*, 2016), and the stony coral *Astroides calycularis* (Casado-Amezú *et al.*, 2012), but less than two other Mediterranean octocorals, *Corallium rubrum* (Ledoux *et al.*, 2010) and *Paramuricea clavata* (Mokhtar-Jamai *et al.*, 2011). In contrast, *E. verrucosa* exhibited the lowest genetic diversity, a finding that may be explained by both biological/ecological and genetic methodology factors: one highly variable locus, Ever009, which exhibited nine alleles when originally developed (Holland *et al.*, 2013a), was excluded from the current analysis due to the presence of null alleles. At the same time, while the low diversity statistics reflect low polymorphism at some *E. verrucosa* loci, reduced polymorphism may itself have been the product of an overall lower level of genetic diversity within the populations studied: at four loci (Ever005, Ever008, Ever011 Ever012) only two or three alleles were detected during initial testing (Holland *et al.*, 2013a), with a maximum of five alleles detected at these loci in the current study. The precise biological/ecological causes of this low genetic diversity (for example, inbreeding, selection) remain to be determined. Overall, differences in the patterns of genetic diversity (*H*_exp_ and *A*_r_) detected between the two species studied were markedly consistent and may, at least in part, be explained by higher genetic connectivity in *A. digitatum*.

*Eunicella verrucosa* has previously been reported as having a low dispersal potential (Munro, 2004); if correct, this would increase the potential for inbreeding. However, the findings of Munro (2004) were based on analysis of only four isoenzymes, markers notorious for their lack of resolution compared to more modern PCR-based techniques (for example, Stevens and Tibayrenc, 1995) and the range and limited number of significant inbreeding coefficients (*F*_IS_) observed for *E. verrucosa* in the current study suggests that the frequency of inbreeding is low, variable between sites and likely due to site-specific factors. Regarding the use of *F*_IS_, while the coefficient is typically referred to as measuring the degree of inbreeding within a population, it actually measures homozygosity excess relative to Hardy-Weinberg expectations, and other processes, for example, the inadvertent combining of data from populations with different allele frequencies (the so called ‘Wahlund effect’) can also drive significant positive *F*_IS_ results. Such a consideration is relevant when seeking to explain the higher number of significant positive *F*_IS_ values obtained for *E. verrucosa* populations (Table 1), as this species showed considerably more evidence of genetic structuring (Figure 2a, Supplementary Appendix S2b) than did *A. digitatum* (Figure 2b, Supplementary Appendix S2d) across the range studied. Thus, given the higher proportion of significant between-population pairwise *F*_ST_s (Supplementary Appendix S2b) observed for *E. verrucosa*, it is possible that cryptic intra-population genetic differentiation may also have played a role in driving significant *F*_IS_ values in this species. If our *F*_IS_ results (especially for *E. verrucosa*) were due to Wahlund effects, such findings would suggest even less inbreeding within the species than the small amount currently postulated. Additionally, the generally low *F*_IS_ values observed also accord with the low proportion of duplicate genotypes detected in *E. verrucosa* (<2%) across the study; the number of *E. verrucosa* individuals at a site with duplicate genotypes ranged from 0 (most samples) to 4 in a small sample (Cam, *N*=11) from north Cornwall. Interestingly, the north Cornwall sample was one of the few samples not collected by our dive teams, and the relatively high proportion of duplicate genotypes at this site may be a reflection of sampling practice rather than biological reality.

For *A. digitatum*, significant *F*_IS_ coefficients were even fewer and lower (though still positive), suggesting only very limited inbreeding in this species; likewise, only a very low proportion (~1%) of all individual *A. digitatum* successfully genotyped had duplicate profiles. While such findings might be expected for broadcast spawning corals (Ayre and Hughes, 2000), exceptions to this pattern are not uncommon; for example, Combosch and Vollmer (2011) studied populations of *Pocillopora damicornis*, a broadcast spawning tropical reef coral, and reported a range of large, mostly positive, significant inbreeding coefficients (*F*_IS_ range: −0.048–0.421), leading them to conclude that widespread inbreeding was apparent in this species in the eastern Pacific.

Compared to previous population genetics studies in octocoral species (for example, Ledoux *et al.*, 2010; Mokhtar-Jamai *et al.*, 2011), the number of significant *F*_IS_ estimates reported here for *E. verrucosa* and *A. digitatum* is globally low: ten significant *F*_IS_ estimates at 27 sites for *E. verrucosa* (Table 1) and four significant *F*_IS_ estimates at 20 sites for *A. digitatum* (Table 2). Overall, such low estimates of *F*_IS_, considered together with the very low numbers of identical individuals sampled in both species, is suggestive of low levels of inbreeding in these two species of octocoral in these parts of their respective ranges.

### Genetic structure and connectivity

Population genetic structure was apparent for *E. verrucosa* at a regional spatial scale (500–2000 km between sample sites), suggesting restrictions to gene flow between populations in different geographical regions. This finding was also supported by analyses of contemporary gene flow (though less so historically), as demonstrated by the limited exchange of genetic material between regions, except for some gene flow between Britain and France (<500 km distance between sites; Figures 3b and 5). Indeed, in both species, analysis of contemporary gene flow suggested that the majority of gene flow occurred between sites within geographical regions, as also observed in *Paramuricea clavata*, a Mediterranean octocoral (Arizmendi-Mejía *et al.*, 2015). In contrast to *E. verrucosa*, little regional structure was apparent in *A. digitatum*, and the only significant differentiation detected was between the samples from the North Sea and those from more westerly areas (>550 km distance between sites); this differentiation appeared to be the product of IBD (as evidenced when comparing the results of IBD analysis with and without North Sea samples of *A. digitatum*; see Figure 4b and Supplementary Figure S5b) and/or a barrier between the samples of western origin and those from the North Sea. Estimates of contemporary effective population size (*N*_e_) were infinite for both species (Supplementary Appendix 6). Assuming these estimates to be accurate, we found no evidence for disequilibrium caused by genetic drift due to a finite number of parents and, thus, any disequilibrium observed was due to sampling error (Waples and Do, 2008). In contrast, estimates of historical effective population sizes were smaller and variable between regions (Supplementary Appendix 6); this result, together with findings from the corresponding analyses of historical and contemporary gene flow (Figure 5) suggest historical patterns of connectivity were not the same as those observed today.

Overall, our findings suggest that *A. digitatum* is panmictic across the western part of the sampled range. One possible explanation for this apparent panmixia is that the winter spawning of *A. digitatum* may facilitate longer dispersal distances via wind-driven currents, thereby increasing genetic connectivity in the eastern Atlantic. Panmixia across similar spatial scales has been reported previously in other marine taxa, including cuttlefish (Wolfram *et al.*, 2006; microsatellite-based study), sea stars (Baus *et al.*, 2005; AFLP-based study), and a closely related species, *Alcyonium hibernicum* (McFadden, 1999; isoenzyme-based study), although in the latter study, in which little or no genetic variation was detected in *A. hibernicum* across the Atlantic, McFadden (1999) also linked her findings to high levels of asexual reproduction by parthenogenesis. Similarly, a recent broad study by Gagnaire *et al.* (2015) highlights the potential impact of large effective population size as an alternative explanation to contemporary panmixia in acting to limit genetic drift, thereby constraining the development of genetic structure, even where gene flow is restricted.

For *E. verrucosa*, populations from Portugal were differentiated from the majority of populations north of the Bay of Biscay. This may represent a natural break in gene flow in which genetic drift either side of the break is the primary driver of population structure; such a conclusion is supported by both the multivariate (PCoA) and Bayesian clustering (STRUCTURE) analyses. This pattern has been reported previously in a number of taxa, including bivalves (Arias *et al.*, 2010), brittlestars (Muths *et al.*, 2009), crustaceans (Papetti *et al.*, 2005; Remerie *et al.*, 2009), microturbellarians (Casu *et al.*, 2011), macroalgae (Neiva *et al.*, 2014) and fish (Milano *et al.*, 2014). However, the significant correlation between genetic and geographic distances in *E. verrucosa* in the current study indicates that a proportion of the differentiation observed is likely explained by IBD. Further analysis omitting the Portugal populations suggested that IBD explains some of the genetic differentiation observed between Portugal and all other populations, but much less of the differentiation observed between Britain, Ireland and France. Interestingly, comparisons with other temperate corals (Table 3) suggest that contemporary patterns of population structure appear often to be driven, at least in part, by IBD, which is possibly due to their sedentary life history and their lack of or shorter pelagic larval duration compared to other benthic marine species. In *E. verrucosa*, analysis of IBD showed no change in significance when the samples from Ireland were removed (Supplementary Appendix 5 and Supplementary Figure S5b), indicating IBD to be a less important driver of population structuring in these Irish samples; such a finding suggests genetic differentiation of these range-peripheral populations is more likely driven by other factors, for example, barriers to gene flow and genetic drift and/or selection. Several previous studies of invertebrates sampled from across this region have also reported genetic differentiation in western Ireland compared to other locations in the northeast Atlantic (Remerie *et al.*, 2009; Casu *et al.*, 2011). These studies attributed this differentiation to recolonisation from relatively northerly refugia that persisted in ice-free coastal areas during the last glacial maximum; however, while Casu *et al.* explained their findings (reduced genetic diversity in more northerly recolonized populations) by reference to founder effects and low numbers of recolonisers (Hewitt, 1996, 1999), Remerie *et al.* postulated the higher genetic diversity and heterogeneity they observed in glaciated areas to be suggestive of range persistence during the last glacial maximum. Our findings for *E. verrucosa* from Ireland (which exhibited the lowest genetic diversity detected in our entire study [*H*_exp_, *A*_r_]) are in line with those of Casu *et al.* (2011) and, likewise, are suggestive of founder effects following post-glacial recolonisation of suitable northerly habitats by small numbers of recolonisers (Nichols and Hewitt, 1994). A lack of sampling at the southern-most limits of the range of *E. verrucosa* also makes it difficult to infer the precise origins of the populations in northwest Ireland, as gaps in our knowledge concerning the genetic identity of all possible source populations limits the accuracy of any putative recolonisation hypotheses. Furthermore, to what degree the contemporary distribution of *E. verrucosa* reflects the extent of the species at the last glacial maximum is unknown, but, to date, its distribution appears not to have extended to areas known to be under ice during the last glacial maximum (Hayward and Ryland, 1995; Hewitt, 1996). In contrast, the distribution of *A. digitatum* in the northeast Atlantic does not show the same pattern and its present day distribution is considerably more northerly, extending from northern Iberia and the Bay of Biscay up to Iceland and Norway (Hayward and Ryland, 1995). Another possible explanation for the differentiation observed in *E. verrucosa* from Ireland in the current study is that the effect of selection may be sufficiently strong in northwest Ireland to mitigate the homogenising effect of gene flow. The populations of *E. verrucosa* found in northwest Ireland are known to be peripheral and inhabit the most northerly limits of the species range (Hayward and Ryland, 1995). Moreover, the lower measures of expected heterozygosity and allelic richness observed in both Irish samples are characteristic of marginal populations, which typically have reduced genetic diversity and can often be under intense selection pressures (Johannesson and André, 2006); our tests for selection identified at least one locus under positive selection. At this stage, however, we do not know which selection pressures, if any, may be acting on these most northerly populations of pink sea fan.

In contrast to the patterns observed between regions, our findings for both octocoral species suggested high gene flow and/or large effective population sizes within regions. For example, for *E. verrucosa*, little differentiation was observed between the two most distant populations within Britain (Sawtooth and Skomer), implying that the transfer of genetic material can potentially occur up to distances of ~480 km. For *A. digitatum*, gene flow was evident at an even larger spatial scale, suggesting that genetic material can be transferred greater distances, potentially more than 1050 km (Payne’s Rock—Norfolk). These results suggest that genetic connectivity is high at an intra-regional scale in both species. However, as observed in many marine species with similar life history traits, large effective population size can also act to reduce (or eliminate) genetic structure, sometimes even *in situations* were gene flow is limited (Gagnaire *et al.*, 2015). Thus, in postulating high gene flow within regions, we need to be mindful of the potential effects of large effective population sizes on genetic structure (or lack of) in these two species.

The hierarchical analysis of *E. verrucosa* population structure revealed a small degree of genetic differentiation between populations in southwest Britain and northwest France at a distance (~200 km) less than that separating some British populations; however, minimal differentiation was evident for *A. digitatum* across this area. The effects of mid-channel currents and local near-shore eddies (Dauvin, 2012) on cross-channel larval migration remains to be explored, although previous research has identified a potential genetic break around western Brittany in a number of taxa, including polychaetes (Jolly *et al.*, 2005), nematodes (Wielgoss *et al.*, 2008) and bivalves (Becquet *et al.*, 2012). In this study, the contrast in genetic connectivity across the English Channel may result from differences in the reproductive biology of the two study species. The pelagic larval duration of *E. verrucosa* is not known, however, evidence from this study suggests this could be shorter than the pelagic larval duration for *A. digitatum*.

### Conservation implications and MPAs

*Eunicella verrucosa* has been listed under the IUCN red list Vulnerable A1d category since 1996, and is recognised as a species facing a high- to medium-term extinction risk due to exploitation (International Union for Conservation of Nature and Natural Resources (IUCN), 2017). It is also listed as a priority species under the UK Biodiversity Action Plan, the UK response to the prevention of biodiversity loss called for by the 1992 Convention on Biological Diversity; in the Republic of Ireland, France and Portugal it does not currently receive any additional protection beyond its IUCN listing. Several of the MCZs recently designated around southwest Britain (for example, Chesil Beach and Stennis Ledges, The Manacles, and The Isles of Scilly) specifically identify *E. verrucosa* as a Protected Feature in their designation listing, and 60% of *E. verrucosa* colonies recorded by diver surveys in southwest Britain fall within areas protected by various other European Union legislation (Pikesley *et al.*, 2016). However, not all of these areas are protected from bottom trawling (for example, The Manacles, Whitsand Bay, Chesil Beach and Stennis Ledges MCZs), suggesting that a large proportion of *E. verrucosa* in Britain remains vulnerable to anthropogenic disturbance and the current level of protection of UK marine ecosystems afforded by the MCZ network is generally insufficient (for example, Lieberknecht and Jones, 2016; Pikesley *et al.*, 2016). Moreover, while the UK government appears to have moved away from the recommended ecological network guidelines for MCZ designation (Lieberknecht and Jones, 2016), the *E. verrucosa* data presented here highlight interesting findings relative to the conservation of ecologically important and prevalent sessile taxa at local (that is, single-site MPAs) to regional (that is, connected metapopulation) scales.

More specifically, the genetic distinctiveness of *E. verrucosa* populations from Ireland underpins an argument for protecting particular sites. Marginal populations often contain rare alleles (the highest extent of private alleles were found at these sites), but may recruit more slowly, and may be genetically isolated, implying vulnerability and reduced resilience (Sanderson, 1996) and therefore an increased need for protection. However, away from the edges of the species range, our data suggest that connectivity can be maintained between populations of these species in some designated MCZs. Moreover, the range of *E. verrucosa* in Britain is small compared to its (primarily Lusitanean) global distribution and, although contemporary connectivity between British populations appears to be a high, at regional spatial scales it could be argued that the genetic distinction of these populations, coupled with their possible role as source populations that act to maintain broader connectivity across this area of the northeast Atlantic, may be sufficient to warrant international conservation efforts (for example, OSPAR Convention, 2013).

In the UK, *A. digitatum* has no specific protective status, is not at the periphery of its global range, and, in our study (apart from the North Sea—English Channel/eastern Atlantic divide), it exhibited relatively high genetic diversity with little evidence of any major barriers to gene flow. Overall, coupled with the high prevalence of this species in UK waters, these factors imply that this species may be a low priority for protection in its own right, and it is likely to receive only patchy, incidental protection based upon the location of current MCZs designated on the basis of other features, although arguments for consideration of this species in design guidelines could still fit both the ‘representativity’ and ‘replication’ principles (Natural England, 2010). However, reduced heterozygosity and impaired sexual reproduction have been reported in another cnidarian species subjected to trawling damage (Henry and Kenchington, 2004) and reduced colony numbers and size have been reported for *A. digitatum* in Lyme Bay, southern England, in trawled areas (Hinz *et al.*, 2011); therefore, this species may be locally vulnerable. Certainly, the occurrence of damaged, sessile populations in disturbed areas, are a useful proxy to highlight degraded ecosystems that may also contain more directly threatened species.

The results from the present study suggest that populations of *E. verrucosa* would benefit from protection across the species range as a connected metapopulation. Although implementing protective measures for a single species across its entire UK range is highly unlikely given commercial and economic pressures within the region, our study serves to highlight areas for consideration in an ecosystem-based management approach. Irish populations of *E. verrucosa* may warrant protection because of their marginality, yet they are not currently protected within the Republic of Ireland beyond their IUCN listing. Analysis of gene flow for both octocoral species studied suggests populations in southwest Britain act as a source for surrounding regions, highlighting the value in protecting these populations. In the UK, the current recommendation for the spacing of designated MPAs is in the region of 40–80 km (Natural England, 2010). In light of our findings, it appears that the distances between these MPAs would generally be sufficient to maintain genetic connectivity of these two octocoral species in UK waters. Of course, this assumes that contemporary local oceanic currents are able to facilitate the transport of enough larvae in each species, whether by a continuing stepping-stone process or a single dispersal event. Managing effective conservation of marine species with overlapping generations and high levels of clonality, such as sponges and corals, can be challenging because characteristic genotypes may persist for decades to centuries, even after significant barriers to gene flow arise. As a result, traditional *F*-statistics may not always represent current patterns of genetic connectivity (Botsford *et al.*, 2009) and these factors should be incorporated when including genetic data into MPA network designation. Furthermore, because of the challenges associated with genotyping octocorals, such as the slow rate of mitochondrial evolution (McFadden *et al.*, 2010) and the difficulty of isolating microsatellites (Liu *et al.*, 2005), the type and numbers of molecular marker used may not be powerful enough to detect a signal of fine-scale population structure. As seen in the current study, while relatively strong patterns of regional structure were detected in *E. verrucosa*, except for some weak structuring between French and English samples of *E. verrucosa* in the Channel, no fine-scale structure (<200 km between sample sites) was detected for either of the species studied. This may be indicative of genetic connectivity between these populations, but could also represent a lack of power in the genetic markers used. Exploration of alternative marker systems may deliver improved resolution (for example, Shinzato *et al.*, 2015) and should prove valuable for future conservation research and the management of MPA networks.

In conclusion, genetic diversity appears to be uniform across the range studied in both species; however, genetic diversity was low in *E. verrucosa*, whereas in *A. digitatum*, it was slightly higher, but still lower than that reported for two species of Mediterranean octocorals (Ledoux *et al.*, 2010; Mokhtar-Jamai *et al.*, 2011). For both species, only limited inbreeding was apparent, and whether this has an impact on fitness and long-term resilience of the populations in question is currently unknown. Regional population structure was identified in *E. verrucosa*, indicative of departures from panmixia at large spatial scales; in contrast, in *A. digitatum*, apart from some genetic differentiation between populations from the North Sea and those from the English Channel/eastern Atlantic, we found little population structure, suggesting high gene flow and connectivity in this species in the western part of the range sampled. Contemporary and historical estimates of effective population size were contrasting and generally difficult to interpret, and for both species the potential role of large *N*_e_s in masking a lack of gene flow cannot be ruled out. Patterns of gene flow were complex, but indicated Britain as a source of genetic variants for both species. Several populations of both species are represented in the UK MPA network and, given the ecological importance of both species, continued monitoring and assessment of their genetic diversity within and beyond protected sites could be a useful measure of the efficacy of the existing network, and a valuable guide to the designation of new MCZs.

## Data archiving

Microsatellite genotypes for *Eunicella verrucosa* and *Alcyonium digitatum* are available from the Dryad Digital Repository: http://dx.doi.org/10.5061/dryad.nj0v4.

## Figures and Tables

**Table 1:** Sampling information and summary statistics for *Eunicella verrucosa* samples

*Region/Population*	*Code*	*N*	*N*_g_	*Depth (m)*	*Lat*	*Long*	*H*_exp_	*A*_r_	*PA*_r_	*F*_IS_
*Britain*										
aIsles of Scilly, Flat Ledge	Fla	23	23	30	49.97	−6.26	0.392	2.45	0.017	−0.021
aIsles of Scilly, Lion Rock	Lio	22	22	24	49.98	−6.31	0.435	2.66	0.017	0.016
aLundy Island	Lun	23 (1)	22	23	51.17	−4.69	0.428	2.61	0.038	0.032
bLyme Bay, The Heroine Wreck	Her	9	9	25	50.68	−2.94	0.432	2.79	0.044	0.006
bLyme Bay, Sawtooth Ledges	Saw	12	12	22	50.68	−2.80	0.383	2.43	0.023	0.106
bLyme Bay, West Tennents Reef	Wte	45 (2)	43	23	50.65	−2.96	0.452	2.74	0.028	0.052
aManacles, Raglan Rocks	Rag	44 (1)	43	28	50.04	−5.04	0.438	2.64	0.036	0.017
aManacles, SS Mohegan Wreck	Moh	30	30	26	50.05	−5.04	0.409	2.52	0.015	**0.140**
Porthallow Bay, Volnay Wreck	Vol	24	24	21	50.07	−5.00	0.401	2.51	0.023	0.002
aPadstow, Camel Estuary	Cam	11 (3)	7	n/a	50.59	−4.95	0.433	2.71	0.001	**−0.202**
Plymouth, Bovisand	Bov	40	40	10	50.34	−4.13	0.423	2.54	0.020	**0.086**
Plymouth, Hand Deeps	Han	36	36	25	50.21	−4.34	0.459	2.76	0.018	0.062
Plymouth, Mewstone	Mew	45 (1)	44	24	50.30	−4.11	0.451	2.65	0.027	0.045
aSkomer Island	Sko	39	39	22	51.74	−5.30	0.445	2.61	0.013	−0.013

*Ireland*										
Donegal, Black Rock	Bla	29	29	25	54.58	−8.43	0.367	2.38	0.034	−0.013
Sligo, Thumb Rock	Thu	48	48	20	54.47	−8.44	0.376	2.46	0.071	**0.097**

*France*										
Brittany, Rade de Brest	Bre	43	43	35	48.31	−4.42	0.412	2.55	0.026	0.047
Brittany, Laonegued Taer	Lao	40	40	30	47.73	−4.06	0.419	2.56	0.059	**0.082**
Brittany, Men Goe	Men	43	43	30	47.69	−3.99	0.418	2.54	0.035	0.055
Brittany, Roscoff1	Ros1	40	40	35	48.75	−3.96	0.419	2.56	0.024	0.014
Brittany, Roscoff2	Ros2	39 (3)	36	25	48.71	−3.90	0.448	2.68	0.049	**0.071**

*Portugal*										
Algarve, Portimao1	Por1	42	42	17	37.10	−8.58	0.429	2.63	0.038	**0.128**
Algarve, Portimao2	Por2	36 (1)	35	18	37.10	−8.56	0.435	2.63	0.058	**0.105**
Algarve, Armacao de Pera1	Arm1	27	27	28	37.09	−8.35	0.402	2.53	0.041	**0.096**
Algarve, Armacao de Pera2	Arm2	44 (1)	43	21	37.05	−8.35	0.392	2.47	0.025	0.028
Algarve, Armacao de Pera3	Arm3	44 (3)	41	25	37.04	−8.36	0.406	2.52	0.039	0.067
Algarve, Faro	Far	44	44	17	36.98	−7.99	0.402	2.53	0.033	**0**.**091**

Abbreviation: CI, confidence interval.

Number of individuals genotyped per population (*N*) (with number of duplicate genotypes), number of unique genotypes per population (*N*_g_), expected heterozygosity (*H*_exp_), allelic richness (*A*_r_), private allelic richness (*PA*_r_) and the inbreeding coefficient (*F*_IS_) are reported for each population. *F*_IS_ values significantly different from zero (95% CI) are highlighted in bold.

a Marine Conservation Zone.

b Candidate Special Area of Conservation.

**Table 2:** Sampling information and summary statistics for *Alcyonium digitatum* samples

*Region/Population*	*Code*	*N*	*N*_g_	*Depth (m)*	*Lat*	*Long*	*H*_exp_	*A*_r_	*PA*_r_	*F*_IS_
*Britain*										
aIsles of Scilly, Seven Stones Reef	Sev	40	40	35	50.03	−6.12	0.624	4.12	0.162	**0.055**
aIsles of Scilly, Trenemene Reef	Tre	42	42	32	49.87	−6.39	0.598	4.07	0.133	0.003
aLundy Island	Lun	36	36	23	51.20	−4.68	0.618	4.15	0.098	0.023
bLyme Bay, Frognor Wreck	Fro	18	18	34	50.53	−2.55	0.626	4.25	0.193	0.042
bLyme Bay, UB74 Wreck	Ub74	19	19	34	50.53	−2.56	0.635	4.18	0.130	0.034
aManacles, Carn-du-rocks	Cdr	35 (2)	33	26	50.05	−5.05	0.636	4.21	0.122	0.007
Porthallow Bay, Volnay Wreck	Vol	28	28	21	50.07	−5.00	0.659	4.31	0.099	−0.001
aSkomer Island, The Lucy Wreck	Luc	23 (1)	22	35	51.74	−5.28	0.594	4.01	0.075	0.027
aSkomer Island, Payne’s Rock	Pay	51	51	30	51.74	−5.31	0.637	4.17	0.109	0.025
aSkomer Island, Tusker Rock	Tus	21	21	29	51.74	−5.26	0.629	4.22	0.189	0.031
Swanage, Betsy Anna Wreck	Bet	26 (2)	24	23	50.62	−1.83	0.620	4.07	0.085	0.024
North Sea, Humberside	Hum	27	27	25	53.64	1.55	0.618	4.08	0.120	−0.014
North Sea, Norfolk	Nor	33	33	25	53.28	1.58	0.668	4.29	0.106	0.059

*Ireland*										
Mayo, Inishturk Island	Ini	48	48	27	53.72	−10.12	0.625	4.13	0.118	0.038
Sligo, Thumb Rock	Thu	18	18	15	54.47	−8.44	0.655	4.24	0.156	−0.058

*France*										
Brittany, Rade de Brest	Bre	43	43	35	48.34	−4.58	0.645	4.23	0.134	**0.068**
Brittany, Laonegued Taer	Lao	29	29	30	47.73	−4.06	0.595	3.99	0.133	0.053
Brittany, Men Goe	Men	35 (1)	34	30	47.69	−3.99	0.653	4.22	0.087	**0.063**
Brittany, Roscoff1	Ros1	41	41	35	48.75	−3.96	0.635	4.23	0.199	0.035
Brittany, Roscoff2	Ros2	42 (1)	41	25	48.71	−3.90	0.649	4.34	0.117	**0.062**

Abbreviation: CI, confidence interval.

Number of individuals genotyped per population (*N)* (with number of duplicate genotypes), number of unique genotypes per population (*N*_g_), expected heterozygosity (*H*_exp_), allelic richness (*A*_r_), private allelic richness (*PA*_r_) and the inbreeding coefficient (*F*_IS_) are reported for each population. *F*_IS_ values significantly different from zero (95% CI) are highlighted in bold.

a Marine Conservation Zone.

b Candidate Special Area of Conservation.

**Table 3:** Summary table comparing the two octocoral species in the current study with previous studies of temperate corals that used microsatellite markers to investigate genetic diversity and population structure in the northeast Atlantic (Atl) and the Mediterranean (Med)

*Species information*				*Study information*						
*Species*	*Reproduction*	*PLD*	*Clonality*	*Sea S; N*	*No. loci*	*Mean H*_exp_	*Mean A*_r_	*Structure*	*IBD*	*References*
*Soft coral*										
*Alcyonium digitatum*	Broadcast	Lecithotrophic, unknown	Yes	Atl 20; 648	8	0.63	4.18	No, *F*_ST_=0.003	Weak, *r*^2^=0.05	This study; Hartnoll, 1975
*Eunicella verrucosa*	Broadcast	Lecithotrophic, unknown	Limited	Atl 27; 905	13	0.42	2.58	Yes, *F*_ST_=0.012	Yes, *r*^2^=0.35	This study; Munro, 2004
*Eunicella singularis*	Brooder	Unknown	Unknown	Med 13; 301	6	0.53	3.58	Yes, NA	NA	Costantini *et al.*, 2016
*Eunicella cavolini*	Unknown	Unknown	Unknown	Med 19; 584	7	0.56	4.24	Yes, *F*_ST_=0.130	Yes, *r*^2^=0.57	Masmoudi *et al.*, 2016
*Corallium rubrum*	Brooder	Lecithotrophic, 4–12 days	Limited	Med 40; 1222	10	0.74	7.30	Yes, *F*_ST_=0.097	Yes, *r*^2^=0.15	Ledoux *et al.*, 2010
*Paramuricea clavata*	Surface brooder	Lecithotrophic, 6–23 days	Unknown	Med 39; 1114	6	0.74	6.48	Yes, *F*_ST_=0.116	Yes, *r*^2^=0.51	Mokhtar-Jamai *et al.*, 2011

*Stony coral*										
*Astroides calycularis*	Brooder	Unknown	Unknown	Med 16; 381	13	0.55	4.38	Yes, *F*_ST_=0.236	Yes, *r*^2^=0.45	Casado-Amezú *et al.*, 2012

Abbreviations: *A*_r_, allelic richness; *H*_exp_, expected heterozygosity; *IBD*, isolation by distance; *N*, number of individuals genotyped; NA, information not available; *PLD*, pelagic larval duration; *S*, number of sites.
