# Supplementary material for: Contrasting patterns of population structure and gene flow facilitate exploration of connectivity in two widely distributed temperate octocorals
Source: Heredity (Edinb). 2017 Mar 15;119(1):35–48. doi: 10.1038/hdy.2017.14 (PMC5520136; doi:10.1038/hdy.2017.14)
Supplement: Supplementary Figure S1 [file hdy201714x1.doc]

**Figure S1:** LOSITAN selection tests under the infinite alleles model for *Eunicella verrucosa* using (a) 13 loci, and (b) excluding locus Ever013, and (c) for *Alcyonium digitatum* using eight loci.

(a)

(b)

(c)
