# Supplementary material for: Contrasting patterns of population structure and gene flow facilitate exploration of connectivity in two widely distributed temperate octocorals
Source: Heredity (Edinb). 2017 Mar 15;119(1):35–48. doi: 10.1038/hdy.2017.14 (PMC5520136; doi:10.1038/hdy.2017.14)
Supplement: Supplementary Figure S3 [file hdy201714x3.doc]

**Figure S3**: AMOVA results table. Significance was assessed using 10,000 permutations.

| **Source of variation** | **Sum of squares** | **Variance components** | **Percentage variation** | **Φ statistic** |
| --- | --- | --- | --- | --- |
| *E. verrucosa* |  |  |  |  |
| Among groups | 55 | 0.039 | 1.58 | 0.016*** |
| Among populations  within groups | 62 | 0.004 | 0.17 | 0.002 |
| Within populations | 4174 | 2.451 | 98.25 | 0.017*** |
| Total | 4291 | 2.495 |  |  |
| *A. digitatum* |  |  |  |  |
| Among groups | 12 | 0.004 | 0.14 | 0.001* |
| Among populations  within groups | 49 | 0.009 | 0.33 | 0.003** |
| Within populations | 3164 | 2.567 | 99.53 | 0.005*** |
| Total | 3225 | 2.579 |  |  |

**p*<0.05, ***p*<0.01, ****p*<0.001
