# Supplementary material for: Contrasting patterns of population structure and gene flow facilitate exploration of connectivity in two widely distributed temperate octocorals
Source: Heredity (Edinb). 2017 Mar 15;119(1):35–48. doi: 10.1038/hdy.2017.14 (PMC5520136; doi:10.1038/hdy.2017.14)
Supplement: Supplementary Figure S4 [file hdy201714x4.doc]

**Figure S4:** Probable number of populations (*K*) for (a) *Eunicella verrucosa*, (b) hierarchical *E. verrucosa*, and (c) *Alcyonium digitatum*. A = mean L(*K*) statistic, D= delta *K* statistic.

(a)

**
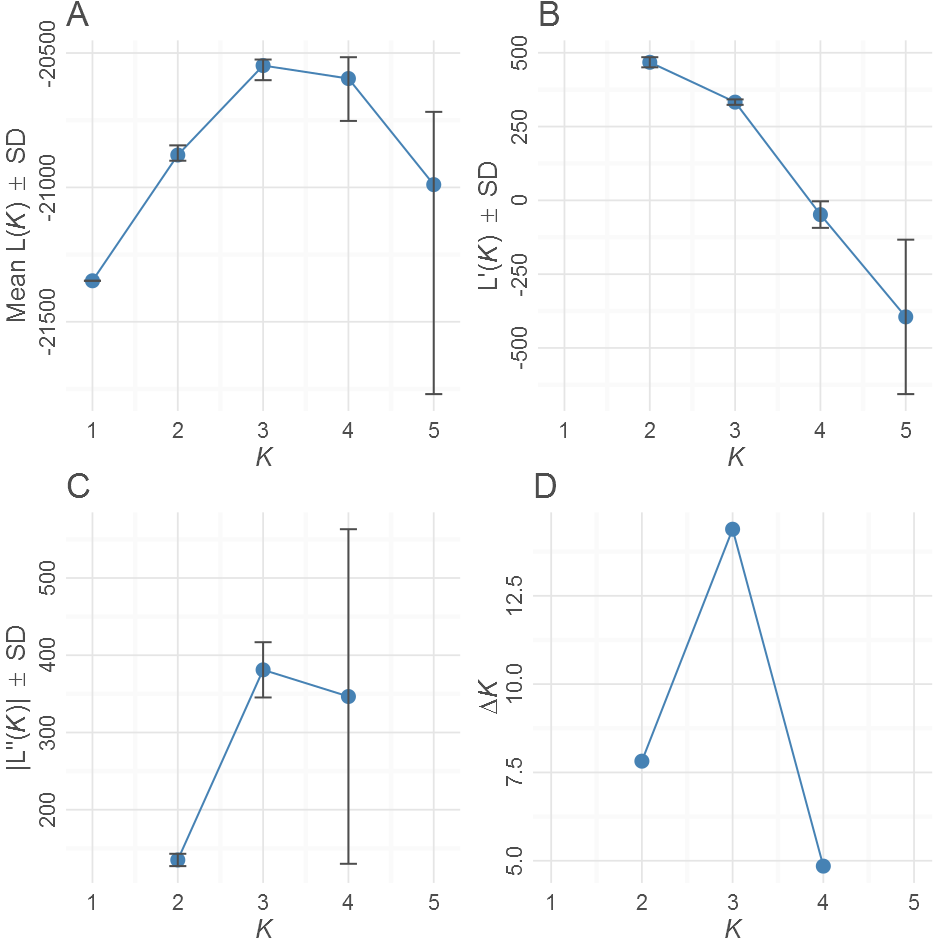
**

(b)

**
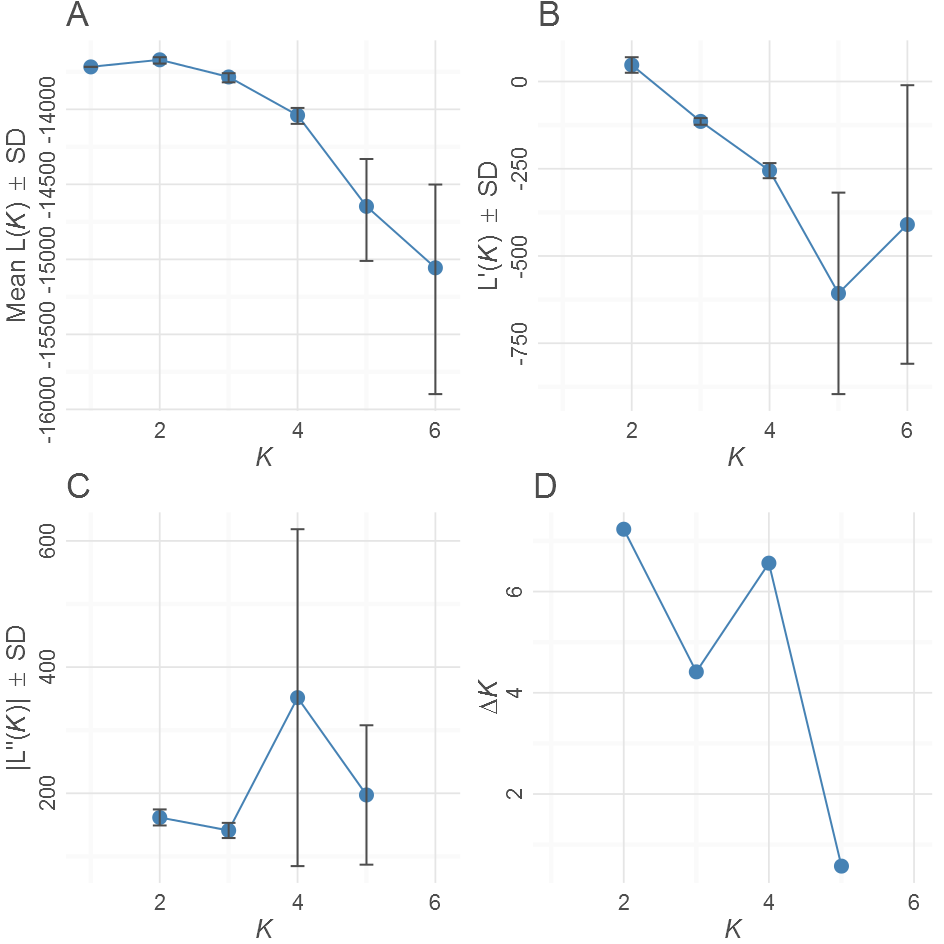
**

(c)

**
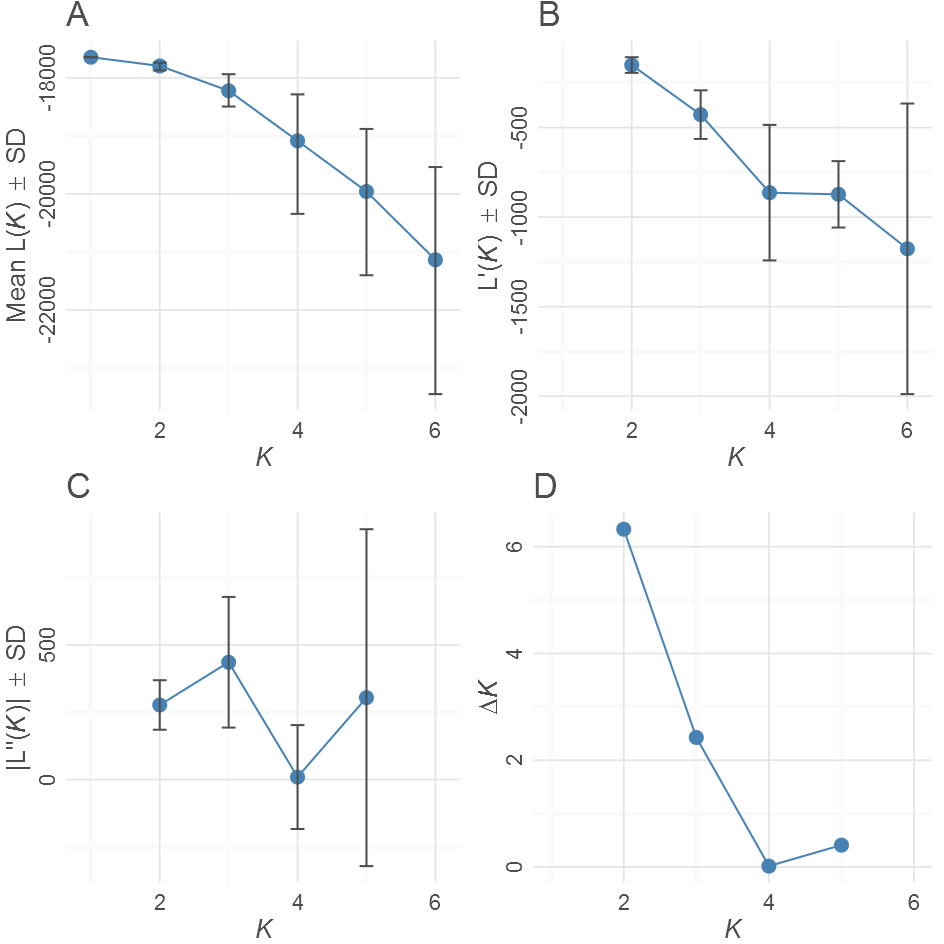
**
