# Supplementary material for: Contrasting patterns of population structure and gene flow facilitate exploration of connectivity in two widely distributed temperate octocorals
Source: Heredity (Edinb). 2017 Mar 15;119(1):35–48. doi: 10.1038/hdy.2017.14 (PMC5520136; doi:10.1038/hdy.2017.14)
Supplement: Supplementary Figure S5 [file hdy201714x5.doc]

**Figure S5:** Isolation by distance (IBD) plots for *Eunicella verrucosa* excluding (a) the Portugal populations, and (b) the Portugal and Ireland populations. (c) IBD plot for *Alcyonium digitatum* excluding the North Sea populations. IBD plot using G_ST_ as the genetic distance (including all populations) for (d) *Eunicella verrucosa* and (e) *Alcyonium digitatum*.

(a)

(b)

(c)

(d)

(e)
