# Supplementary material for: Contrasting patterns of population structure and gene flow facilitate exploration of connectivity in two widely distributed temperate octocorals
Source: Heredity (Edinb). 2017 Mar 15;119(1):35–48. doi: 10.1038/hdy.2017.14 (PMC5520136; doi:10.1038/hdy.2017.14)
Supplement: Supplementary Figure S6 [file hdy201714x6.doc]

**Figure S6**: (a) Historical gene flow (*M*) and effective population size (*N*_e_) estimates for *Eunicella verrucosa* and *Alcyonium digitatum* using Migrate-n. Historical *N*_e_ was calculated using the equation: *N*_e_ = theta/4µ, assuming a mutation rate (µ) of 10^-4^ per locus per generation. (b) Contemporary gene flow and effective population size (*N*_e_) estimates for *Eunicella verrucosa* and *Alcyonium digitatum* using BayesAss. Source populations = rows, sink populations = columns. Group colours match those used in Figs 2, 3 and 5.

(a)

| ***E. verrucosa*** | Historical gene flow (*M*) | | | |  |  |
| --- | --- | --- | --- | --- | --- | --- |
| **Group** | Britain | Ireland | France | Portugal | Theta | *N*_e_ |
| Britain | **-** | 164.5 | 61.9 | 48.2 | 4.311 | 10,778 |
| Ireland | 2.9 | **-** | 15.1 | 15.2 | 2.478 | 6,195 |
| France | 11.0 | 19.3 | **-** | 21.4 | 2.119 | 5,298 |
| Portugal | 10.0 | 51.6 | 33.4 | **-** | 1.859 | 4,648 |
|  |  |  |  |  |  |  |
| ***A. digitatum*** | Historical gene flow (*M*) | | | |  |  |
| **Group** | Britain | Ireland | France | North Sea | Theta | *N*_e_ |
| Britain | **-** | 85.1 | 27.5 | 52.1 | 0.555 | 1,388 |
| Ireland | 6.5 | - | 7.6 | 25.7 | 30.549 | 76,373 |
| France | 21.2 | 49.9 | **-** | 20.0 | 1.184 | 2,960 |
| North Sea | 9.6 | 31.6 | 19.7 | **-** | 27.554 | 68,885 |

(b)

| ***E. verrucosa*** | Contemporary gene flow | | | |  |
| --- | --- | --- | --- | --- | --- |
| **Group** | Britain | Ireland | France | Portugal | *N*_e_ |
| Britain | 0.9907 | 0.0123 | 0.2834 | 0.0085 | ∞ |
| Ireland | 0.0017 | 0.9647 | 0.0024 | 0.0031 | ∞ |
| France | 0.0030 | 0.0098 | 0.6774 | 0.0085 | ∞ |
| Portugal | 0.0046 | 0.0133 | 0.0368 | 0.9799 | ∞ |
|  |  |  |  |  |  |
| ***A. digitatum*** | Contemporary gene flow | | | |  |
| **Group** | Britain | Ireland | France | North Sea | *N*_e_ |
| Britain | 0.9724 | 0.2965 | 0.3207 | 0.3004 | ∞ |
| Ireland | 0.0012 | 0.6716 | 0.0019 | 0.0052 | ∞ |
| France | 0.0251 | 0.0266 | 0.6752 | 0.0223 | ∞ |
| North Sea | 0.0014 | 0.0052 | 0.0021 | 0.6720 | ∞ |
